# Comparing machine learning algorithms for multimorbidity prediction: An example from the Elsa-Brasil study

**DOI:** 10.1371/journal.pone.0275619

**Published:** 2022-10-07

**Authors:** Daniela Polessa Paula, Odaleia Barbosa Aguiar, Larissa Pruner Marques, Isabela Bensenor, Claudia Kimie Suemoto, Maria de Jesus Mendes da Fonseca, Rosane Härter Griep

**Affiliations:** 1 National School of Statistical Sciences, Brazilian Institute of Geography and Statistics, Rio de Janeiro, Brazil; 2 Institute of Nutrition, University of the Rio de Janeiro State, Rio de Janeiro, Brazil; 3 National School of Public Health, Oswaldo Cruz Foundation, Rio de Janeiro, Rio de Janeiro, Brazil; 4 Department of Internal Medicine, Faculdade de Medicina da Universidade de São Paulo & Hospital Universitário, Universidade de São Paulo, São Paulo, Brazil; 5 Division of Geriatrics, Department of Clinical Medicine, Faculdade de Medicina, Universidade de São Paulo, São Paulo, Brazil; 6 Department of Epidemiology, National School of Public Health (ENSP/Fiocruz), Rio de Janeiro, Brazil; 7 Health and Environmental Education Laboratory, Oswaldo Cruz Institute (IOC), Rio de Janeiro, Brazil; National University of Sciences and Technology, PAKISTAN

## Abstract

**Background:**

Multimorbidity is a worldwide concern related to greater disability, worse quality of life, and mortality. The early prediction is crucial for preventive strategies design and integrative medical practice. However, knowledge about how to predict multimorbidity is limited, possibly due to the complexity involved in predicting multiple chronic diseases.

**Methods:**

In this study, we present the use of a machine learning approach to build cost-effective multimorbidity prediction models. Based on predictors easily obtainable in clinical practice (sociodemographic, clinical, family disease history and lifestyle), we build and compared the performance of seven multilabel classifiers (multivariate random forest, and classifier chain, binary relevance and binary dependence, with random forest and support vector machine as base classifiers), using a sample of 15105 participants from the Brazilian Longitudinal Study of Adult Health (ELSA-Brasil). We developed a web application for the building and use of prediction models.

**Results:**

Classifier chain with random forest as base classifier performed better (accuracy = 0.34, subset accuracy = 0.15, and Hamming Loss = 0.16). For different feature sets, random forest based classifiers outperformed those based on support vector machine. BMI, blood pressure, sex, and age were the features most relevant to multimorbidity prediction.

**Conclusions:**

Our results support the choice of random forest based classifiers for multimorbidity prediction.

## Introduction

Multimorbidity, usually defined as the presence of two or more chronic diseases, represents a huge challenge for health systems all over the world [1, 2]. People with multiple chronic diseases commonly experienced loss of functional abilities, more frequent and longer hospitalization, and increased risk of premature death [1–3]. Traditional medical practices based on diagnosis and treatment of diseases in isolation highlight the limitations in health systems for the treatment of multimorbidity [4].

Although multimorbidity is a challenge worldwide, there are differences between low and middle-income countries (LMIC´s) and high-income countries (HIC´s) [3, 5, 6]. Multimorbidity affects more young people in LMIC´s than in HIC´s and represents a large part of the burden of disease in those countries [5–7]. The different profiles imply the need for different preventive approaches and continued treatment. In this sense, the knowledge of multimorbidity patterns and factors related, especially the modifiable factors, can help public policies for the prevention and treatment of multiple chronic diseases.

Studies on multimorbidity patterns in low and middle-income countries (LMIC´s) are still sparse [8]. Particularly, some studies have already described the most prevalent dyads and triads of chronic conditions and identified socio-economic factors as related to multimorbidity, but most studies deal with multimorbidity as a count of diseases [9, 10]. Given the diversity of cooccurrences of chronic diseases, the number of diseases does not allow expressing the variety of possible multimorbidity patterns as well as related factors.

Recently, machine learning (ML) methods have been applied to many problems in public health to improve prediction and discover complex patterns of relationships not found by traditional methods [11]. Studies using unsupervised ML techniques have been published for the recognition of multimorbidity patterns, but there is still a gap of studies about modeling and prediction of multiple chronic diseases, possibly due to the complexity of establishing the classification of multiple diseases simultaneously [12–14].

The main challenges for multimorbidity prediction, not yet jointly addressed in the multiple disease prediction scenario, are: to incorporate the relationship among diseases in the prediction; to deal with the imbalanced and low occurrence of diseases; and to find a set of features that are low-cost, to be able to predict with reasonable accuracy the occurrence of multiple chronic conditions [12–17]. The contributions of this work are present a ML approach based on multilabel classifiers to deal with these problems, and apply it to the build and comparison of cost-effective multimorbidity prediction models, using a large sample from the Brazilian Longitudinal Study of Adult Health (ELSA/Brasil). We provided a comparison of the performance of seven multilabel classifiers and identified the features that are most relevant in the prediction.

To encourage the use of multilabel classifiers, we developed a web application available online at https://danielapaula.shinyapps.io/Multilabel_Tool/, where prediction models can be built and used practically and intuitively. The application can be used by the general public since it does not require prior knowledge of machine learning or programming.

## Methods

### Study population

ELSA-Brasil is a multicenter cohort study conducted in six Brazilian capitals. The main objective is to investigate the incidence and risk factors associated with cardiovascular diseases and diabetes. The baseline was performed from 2008 to 2010 and enrolled 15105 active or retired civil servants (35–74 years). Participants were submitted to a set of examinations, in addition to detailed personal interviews by trained personnel. The sampling procedures and study design of ELSA-Brasil have been reported previously [18, 19].

ELSA-Brasil was approved by the National Research Ethics Committee (Conep—No. 976), and the research protocol developed at all Research Centers was approved by the Research Ethics Committee of the Oswaldo Cruz Institute (CEP Fiocruz/IOC—No. 343/06). All participants gave written consent to participate.

### Study variables

#### Outcome variables

Chronic diseases with prevalence greater than or equal to 5% at baseline were included. Multimorbidity was defined as the presence of at least two chronic diseases in the set of 10 morbidities: cancer, diabetes, dyslipidemia, common mental disorder, migraine, heart disease (acute myocardial infarction, angina pectoris or heart failure), asthma, cirrhosis, joint problems, and kidney disease.

Cancer, heart disease, asthma, cirrhosis, joint problems, and kidney disease were identified by the participant’s self-report of a previous diagnosis by a physician. Dyslipidemia was identified by clinical exam. Diabetes was defined by self-report or use of medication and when not reported, it was defined by clinical exam [20–25].

Common mental disorders were identified using the Clinical Interview Schedule—Revised (CIS-R) instrument (cut-off point for the presence of a disorder ≥ 12 points) [26]. Migraine was defined according to a detailed headache questionnaire based on the International Headache Society (IHS) criteria [24].

#### Predictors variables

The choice of the set of predictors was based on previously published predictive models for chronic diseases [3, 26–28]. We considered 31 variables that do not imply additional costs beyond those easily accessible by physicians in a clinical visit. The variables were defined as follows:

Sociodemographic variables: sex, age (in years), education (never attended to school to elementary, secondary, undergraduate and postgraduate), self-reported race/color (based on Brazilian Census: white, “pardo,” black, indigenous and Asian), marital status (single or not single), per capita household income (in dolar), children (yes/no), maternal education (never attended to school, incomplete elementary, elementary, secondary and undergraduate).

Lifestyle and dietary variables: smoking (never, past, or current), consumption of alcohol (not consuming, moderate—consumption <210 and 140 g/wk for men and women, respectively, excessive–higher than previous consumption limits) [29], physical activity in leisure time (weak, moderate, and strong following the classification of the International Physical Activity Questionnaire, in the domain of leisure-time physical activity) [30]. Days of physical activity (number of days/week) [28]. Sleep symptom (yes/no). Sleeping problem (yes/no) [28]. Fruit consumption and vegetable consumption (high, daily, weekly and rarely) [31]. Coffee consumption (no; yes, with caffeine; yes, decaffeinated) [28].

Anthropometric variables: body mass index (BMI) (kg/m^2^), systolic blood pressure (BP) (mmHg), diastolic BP (mmHg), heart rate (BPM), waist-hip ratio.

Family history variables (yes/no): hypertension, diabetes, heart disease, stroke, cerebrovascular accident, angioplasty and stent, sudden death, asthma, cancer.

### Statistical analysis

#### Descriptive analysis

The analytical sample considered 14836 participants who provided information about the chronic diseases considered. Descriptive analysis was performed. Participants were grouped according to the number of chronic conditions (0,1,2,3,4, 5 or more), and differences among the groups were tested by Pearson’s chi-square test, analysis of variance, and Kruskal-Wallis test. The multimorbidity patterns were identified and the prevalence analyzed [32]. The level of significance was set at p < .05. The R 4.1.0 software was used in all analyses.

#### ML algorithms

*i*. *Incorporating the relationship between the diseases*. Multilabel classifiers approach the issue of incorporating the relationship between the diseases in the model design. They provide different ways of establishing the relationship between the diseases and can be divided into two main categories: Algorithm adaptation and problem transformation. In this work, seven multilabel classifiers were implemented and their performances compared. One algorithm adaptation method (multivariate random forest) and three problem transformation methods (Binary Relevance, Dependent Binary Relevance, and Classifier Chain), with support vector machine (SVM) and random forest (RF) as base classifiers for each one of transformation methods [33, 34]. These methods differ in the way they establish the relationship between the diseases for prediction:

*Multivariate random forest (MTV-RF)*–The relationship between the labels is established by a composite normalized Gini index splitting rule, which uses a weighted covariance structure (e.g., auto regressive, compound symmetry) to assign the relationship between the labels.

*Binary relevance (BR)*—The simplest problem transformation method that implements a binary classifier for each label. The labels are predicted independently of each other and label dependencies are not taken into account.

*Dependence Binary relevance (DBR)*—The multilabel classification is transformed into simple binary classifications for each label, as well as in the BR method, but the dependence between the labels is established using for each label the actual information of all binary labels (except the target outcome) as additional features.

*Classifier Chain (CC)*–A binary classifier is trained for each label following a given order. The dependence between the labels is designed by including in the feature space of each classifier the true label information of all previous labels in the chain.

The choice of classifiers was based on scalability for large datasets and performance on prediction problems [17–19].

For model evaluation, we consider the performance measures usually considered for multilabel classifiers: Hamming loss, subset accuracy, accuracy, and F-measure, defined as follows [34]:

Let D be a multilabel dataset, |*D*| the number of observed instances (for example, the number of participants in the study), L the full set of labels in D, and |*L*| the number of labels (for example, the number of diseases considered). For Y_i_ the label set of i-th instance (observed diseases for the i-th participant), and Z_i_ the subset of predicted labels (predicted diseases for i-th participant), we define:

Hamming Loss: It is the most common evaluation metric in the multilabel literature, computed as the symmetric difference between predicted and true labels (predicted diseases that were not observed or observed diseases that were not predicted) divided by the total number of labels.

$$HammingLoss=\frac{1}{\left|D\right|}\sum_{i=1}^{\left|D\right|}\frac{{|Y}_{i}\Delta Z_{i}|}{\left|L\right|}$$ (1)

where Δ is the operator that returns the symmetric difference between *Y*_*i*_, the label set of the i-th instance, and *Z*_*i*_, the predicted one. The |.| operator counts the number of 1’s in this difference, in other words the number of miss predictions.

Subset Accuracy: This metric is also known as 0/1 Subset Accuracy and Classification Accuracy, and it is the most strict evaluation metric. The[[]] denotes de Iverson bracket, which returns 1 if the expression inside it is true or 0 otherwise. In this case, its value is 1 only if the predicted set of labels equals the true one.

$$SubsetAccuracy=\frac{1}{\left|D\right|}\sum_{i=1}^{\left|D\right|}\left[[Y_{i}=Z_{i}]\right]$$ (2)

where [[*Y*_*i*_ = *Z*_*i*_]] returns 1 if all labels for i-th instance are equal to the predicted ones.

Accuracy: It is defined as the proportion of correctly predicted labels concerning the total number of labels (predicted or observed) for each instance.

$$Accuracy=\frac{1}{\left|D\right|}\sum_{i=1}^{\left|D\right|}\frac{{|Y}_{i}\cap Z_{i}|}{\left|Y_{i}\cup Z_{i}\right|}$$ (3)

where |*Y*_*i*_∩*Z*_*i*_| is the number of correctly predicted labels, and |*Y*_*i*_∪*Z*_*i*_| is the total number of active labels, in the both real label set and the predicted one.

F-Measure: This metric is the harmonic mean between Precision and Recall, providing a balanced assessment between precision and sensitivity.

$$F-Measure=2*\frac{Precision*Recall}{Precision+Recall}$$ (4)

where $Precision=\frac{1}{\left|D\right|}\sum_{i=1}^{\left|D\right|}\frac{{|Y}_{i}\cap Z_{i}|}{\left|Y_{i}\right|}$ (5), with |*Y*_*i*_|, the total number of truly relevant labels, and |*Y*_*i*_∩*Z*_*i*_| defined as in Eq (3). $Recall=\frac{1}{\left|D\right|}\sum_{i=1}^{\left|D\right|}\frac{{|Y}_{i}\cap Z_{i}|}{\left|Z_{i}\right|}$ (6), with |*Z*_*i*_|, the total number of predicted labels, and |*Y*_*i*_∩*Z*_*i*_| defined as in Eq (3).

*ii*. *Dealing with imbalance*. To evaluate the imbalance of disease occurrences we used cardinality, density, and IRLbl (Imbalance ratio per Label). These measures characterize the dataset and can influence the classifiers, they are defined as follows [14, 35]:

Let D be a multilabel dataset, |*D*| the number of observed instances (for example, the number of participants in the study), L the full set of labels in D, and |*L*| the number of labels (for example, the number of diseases considered). For Y_i_ the label set of i-th instance (observed diseases for the i-th participant) in D, we define:

Label Cardinality: is the average number of labels of the observations in a dataset D:

$$LC(D)=\frac{1}{\left|D\right|}\sum_{i=1}^{\left|D\right|}{|Y}_{i}|$$ (7)

where |*Y*_*i*_| is the total number of truly relevant labels for the i-th instance.

Label Density: is the average number of labels of the observations in a dataset D divided by |L|:

$$LD(D)=\frac{1}{\left|D\right|}\sum_{i=1}^{\left|D\right|}\frac{{|Y}_{i}|}{\left|L\right|}$$ (8)

IRLbl (Imbalance ratio per Label):

$$IRLbl(y)=\frac{{max}_{y´\in L}(\sum_{i=1}^{\left|D\right|}\left[[y´\in Y_{i}]\right])}{\sum_{i=1}^{\left|D\right|}\left[[y\in Y_{i}]\right]}$$ (9)

where the symbol [[]] denotes the Iverson bracket, which returns 1 if the expression inside it is true or 0 otherwise, *y* denotes the label for which the measure *IRLbl*(*y*) is calculated, *y*´ denotes a label evaluated in L (*y*´∈*L*), and max is the maximal value.

IRLbl is a measure calculated individually for each label and represents the maximum observed occurrence in the set of labels divided by the occurrence of each label (the highest observed occurrence of a disease divided by the occurrence of each disease). The higher is the IRLbl the larger would be the imbalance, allowing to know which labels are in minority or majority. MeanIR is the average IRLbl for an MLD. It is useful to estimate the global imbalance level [14, 35].

Due to the imbalance observed in the dataset regarding the chronic conditions, the performance measures were averaged over stratified 10-fold cross-validation (CV), repeated 5 times. In each iteration, the algorithms were then trained in turn on nine partitions and evaluated on the remaining partition. We consider a nested 3-fold cross-validation for hyperparameter tuning. The methods can handle missing values internally, except for the SVM-based algorithms, for which the imputation was performed within the CV, on the test and training partitions separately, to avoid bias in performance estimation. Technical details are given in the appendix (SMethods1 in S1 Appendix).

A possible strategy to deal with imbalance is to consider a resampling algorithm in model design. To evaluate the impact of resampling algorithms, we applied the random oversample based on the IRLbl [35]. The oversample was applied on each partition of the training set generated during the stratified cross-validation procedure [18, 36, 37].

*iii*. *Searching for low-cost predictors*. To consider searching for low cost features, we used a feature selection technique based on information gain. Variable importance was estimated for the MTV-RF model. We analyzed the effect of the number of features in classification performance using the Binary Relevance + Information Gain (BR+IG) approach [16, 38]. This feature selection consists of first transforming the multilabel data into single-label datasets, by Binary Relevance, and then using them to select features based on information gain scores. We analyze the model performances for different numbers of features following the score ranking. Variable importance was averaged over 100 runs for the MTV-RF using a generalization of the permutation variable importance [33].

*iv*. *Web application*. The web application for building and using multilabel classifiers, called Multilabel_Tool, is available at https://danielapaula.shinyapps.io/Multilabel_Tool/, and was developed using the Shiny package from R. A sample of the R code files for creating the application is available at https://github.com/paula-daniela/Multilabel_Tool.git, as well as two examples of datasets that can be used as input files for building the models and making predictions.

On the developed web application, MTV-RF, BR, and CC classifiers are available, as well as measures of information gain (BR+IG), so that it is possible to evaluate the performance of the models for different sets of features selected from (BR+IG), and to perform hyparameter tuning by cross-validation. A brief tutorial of the web application is available in the appendix (SResults3 in S1 Appendix).

## Results

Among the 14836 participants included in this study, 8347 (56.3%) had multimorbidity (Table 1). The average age was 52.1 years, with a range of 34–75 years. Most of the participants with multimorbidity were women 5060 (60.6%) and had a higher mean age (53.2). Among the subgroups with the highest number of diseases, we observe a gradient of increase in the average age, and proportion of women (Table 1).

**Table 1:** Sociodemographic characteristics for the study population, according to the number of chronic conditions.

Variables	Number of chronic conditions						Total	Missing
	0 (n = 2238)	1 (n = 4251)	2 (n = 4111)	3 (n = 2550)	4 (n = 1163)	5+ (n = 523)	n = 14,836	n (%)
Age, mean (sd)	49.3 (8.6)	51.4 (9)	52.3 (9)	53.2 (9)	54.7 (8.9)	56.2 (8.7)	52.1 (9.1)	0
Sex, n (%)								0
Male	1288 (57.6)	2194 (51.6)	1846 (44.9)	960 (37.6)	367 (31.6)	114 (21.8)	6769 (45.6)	
Female	950 (42.4)	2057 (48.4)	2265 (55.1)	1590 (62.4)	796 (68.4)	409 (78.2)	8067 (54.4)	
Education, n (%)								0
Never attended school to elementary school	257 (11.5)	496 (11.7)	538 (13.1)	323 (12.7)	163 (14)	104 (19.9)	1881 (12.7)	
Secondary school	813 (36.3)	1381 (32.5)	1398 (34)	925 (36.3)	453 (39)	189 (36.1)	5159 (34.8)	
University degree	1168 (52.2)	2374 (55.8)	2175 (52.9)	1302 (51.1)	547 (47)	230 (44)	7796 (52.5)	
Family per capita income, mean (sd)	900 (732.7)	973.2 (803.1)	951.9 (771.3)	931.2 (770.3)	913.2 (786.8)	798.8 (618.4)	938.2 (771.5)	59 (0.4)
Skin color/race, n (%)								171 (1.2)
White	1127 (50.7)	2252 (53.8)	2101 (51.7)	1323 (52.5)	609 (52.9)	231 (44.6)	7643 (52.1)	
Pardo	642 (28.9)	1169 (27.9)	1152 (28.4)	715 (28.4)	294 (25.5)	165 (31.9)	4137 (28.2)	
Black	366 (16.4)	629 (15)	661 (16.3)	394 (15.6)	214 (18.6)	102 (19.7)	2366 (16.1)	
Indigenous	27 (1.2)	33 (0.8)	42 (1.0)	30 (1.2)	12 (1.0)	7 (1.4)	151 (1.0)	
Asian	63 (2.8)	106 (2.5)	105 (2.6)	59 (2.3)	22 (1.9)	13 (2.5)	368 (2.5)	
Marital Status, n (%)								0
Single	638 (28.5)	1357 (31.9)	1413 (34.4)	920 (36.1)	477 (41.0)	222 (42.4)	5027 (33.9)	
Not single	1600 (71.5)	2894 (68.1)	2698 (65.6)	1630 (51.1)	686 (59)	301 (57.6)	9809 (66.1)	

Note. All variables showed significant differences between the subgroups.

**Fig 1:**
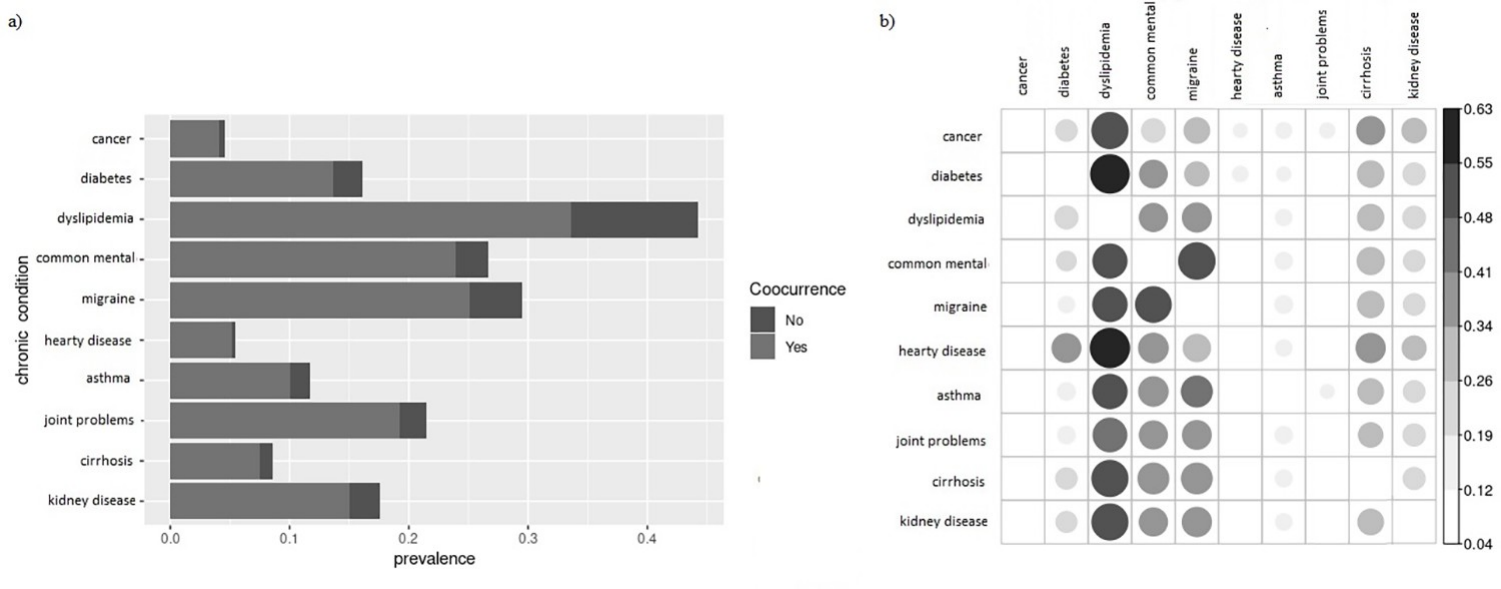
Prevalence and cooccurrence of chronic conditions. a) Prevalence of chronic conditions (light gray + dark gray), occurring jointly with other conditions (light gray) and occurring without any condition (dark gray); b) Prevalence of y-axis comorbidities among participants who have x-axis conditions; common mental–common mental disorder.

The condition with the highest prevalence was dyslipidemia (44.2%), followed by migraine (29.5%) and common mental disorder (26.7%) (Fig 1A). Dyslipidemia, migraine and common mental disorder also had the highest prevalences when with co-occurring conditions (33.6%, 25.1%, and 23.9%, respectively) (Fig 1A). Overall, for all diseases, among the most common co-occurring diseases were dyslipidemia migraine and common mental disorder (Fig 1B). A high prevalence of dyslipidemia was observed among people with heart disease (63%), and with diabetes (61%), high prevalence of migraine among participants with common mental disorder (55%), cirrhosis (41%), asthma (41%), joint problems (36%) and kidney disease (35%) (Fig 1B).

Regarding the imbalance between occurrences, the dataset has a label cardinality of 1.86 and a density of 0.19. The mean IRLbl of the diseases was 3.82, indicating an average imbalance of approximately four between the occurrences of dyslipidemia and the other diseases. To evaluate resampling methods to deal with imbalance we applied random oversampling based on IRLbl for the MTV-RF classifier. Oversampling was applied to each partition of the training set generated during the stratified cross-validation procedure. We observed a slight improvement in all performance measures, especially in subset accuracy, but, due to the computational cost, we did not apply the resampling method to the other classifiers. The results are in the appendix (SResults1 in S1 Appendix).

The performance measures for the ML algorithms, considering all predictors, are presented in Table 2. In general, the methods based on RF, as adaptative as well as transformation, showed better performance than the methods based on SVM. The best performance was achieved for RF-CC.

**Table 2:** Prediction performance measures.

Method	Accuracy	Subset Accuracy	Hamming Loss	F-Measure
MTV-RF	0.335	0.145	0.166	0.407
RF-CC	0.339	0.152	0.165	0.409
SVM-BR	0.322	0.140	0.168	0.392
SVM-DBR	0.313	0.143	0.169	0.380
RF-BR	0.336	0.145	0.165	0.409
RF-DBR	0.328	0.147	0.166	0.397
SVM-CC	0.315	0.145	0.169	0.381

Note. MTV- Multivariate; RF-Random Forest; CC- Classifier Chain; SVM- Support Vector Machine; BR- Binary Relevance; DBR- Dependent Binary Relevance.

**Fig 2:**
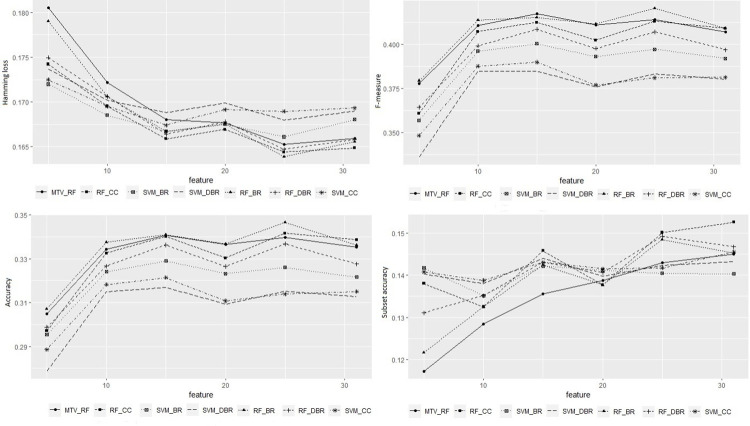
Performance of multilabel classifiers according to the number of features. MTV- multivariate; RF-Random Forest; CC- Classifier Chain; SVM- Support Vector Machine; BR- Binary Relevance; DBR- Dependent Binary Relevance.

For each feature, the info gain was evaluated using the BR+IG approach. BMI had the highest information gain, followed by systolic BP, waist-hip ratio, diastolic BP, and age. Sets of 5, 10, 15, 20, and 25 features were selected by BR+IG rank, to compare the performance of the methods on smaller sets of predictors. For most methods, the performance measures F-measure and accuracy become stable after 10 features, and for Hamming loss, from 15 features on. Regarding subset accuracy, SVM-based classifiers were the most stable, regardless of the number of features (Fig 2).

SVM-based transformation methods perform better concerning Hamming loss and subset accuracy for a few features (Fig 2). As the number of features increases, RF-based methods take the lead, besides performing better regarding accuracy and F-measure, even for small numbers of features. RF-BR is one of the best methods concerning accuracy and F-measure for all feature sets. RF-BR is among the best methods regarding Hamming loss from 15 features, with performance similar to RF-CC and RF-DBR, and concerning subset accuracy, from 22 features. The difference is that the last two methods, which include dependency between labels, have higher subset accuracy than RF-BR, with RF-CC outperforming RF-DBR from 25 features on.

**Fig 3:**
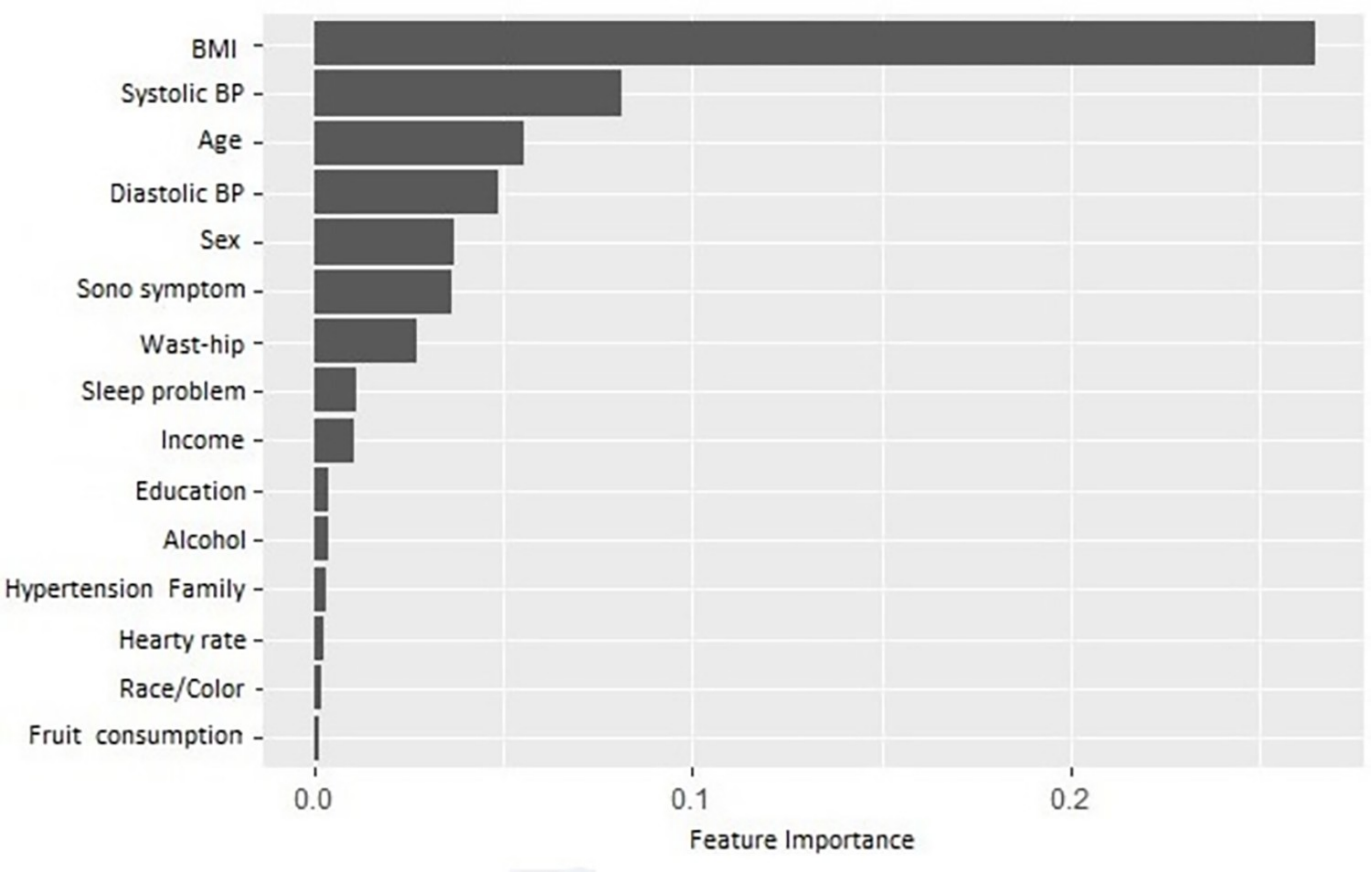
Average variable importance for the first top 15 features using multivariate random forest. BP-Blood Pressure; BMI-Body Mass Index.

Analogously to the BR+IG rank, the variable importance for the MTV-RF showed that BMI was the best predictor for multimorbidity, followed by systolic BP, age, diastolic BP, and sex (Fig 3). Furthermore, the sets of the first top eight predictors were equal by both importance and BR+IG ranks. The importance decreases for the other features with values close to zero.

To exemplify the classification rule and interpretability of the MTV-RF classifier, a subsample of the study population was considered (n = 1340), for simplicity. The resulting classification rule and the trees generated for each disease considered are available in the appendix (SResults2 in S1 Appendix).

## Discussion

Recent studies on multimorbidity have implemented ML techniques to identify patterns of association between chronic conditions [11–13]. However, knowledge about modeling and prediction is still limited. Few studies deal with predictions for multiple diseases, mostly used counts [9, 10, 39]. When predicting with counting, the full complexity of relationships between diseases and between diseases and predictors is neglected in exchange for a less complex modeling process. The difficulties in predicting multimorbidity and how to overcome these issues when building a classifier have not yet been reported. The present study is the first to present the main problems in predicting multiple diseases and how to solve them, besides providing a user-friendly web application for building and using prediction models.

We present a machine learning approach based on multilabel classifiers, and use it to build and compare the performance of seven classifiers for predicting multimorbidity. This methodology and developed web application can be used to build feasible models, for screening individuals with multimorbidity from a small set of characteristics easily accessible in clinical practice. In the context of LMIC´s, with scarce resources, where it is known that multimorbidity represents a high burden of disease, these models can help as a tool for prevention and targeting of interventions based on the modifiable factors [7, 8]. Moreover, since the models can predict the disease’s cooccurrence, they can support the design of integrated clinical practices of care instead of conventional fragmented strategies for each chronic disease, one of the main challenges for multimorbidity prevention and management [8].

The prevalence of multimorbidity observed in this study (56.3%) was higher than expected in Brazil (reported in previous studies between 16.8% and 24.2%) [10, 40–43]. Differences between prevalences can occur due to several reasons, such as the different sets of diseases and differences in variables such as age. The majority of participants with multimorbidity were women and had higher average age. Among participants with at least two chronic conditions, we observed a gradient of increase in the proportion of people with primary or secondary education, and decrease in average per capita income. Most studies found that age is a risk factor for multimorbidity as well as sex, however, there is still no consensus on other factors such as education and income [8].

Dyslipidemia, migraine and common mental disorder were the most prevalent diseases and were also among the most cooccurring. High cooccurrences were also found between diabetes and dyslipidemia, heart disease and dyslipidemia, migraine and common mental disorder, asthma and migraine, and joint problems and migraine. These results are in agreement with previous studies that reported a high prevalence of dyslipidemia, as well as associations between mental disorders, joint problems, migraine, and respiratory diseases [8, 41–45]. The patterns of association found are compatible with the patterns previously identified in Brazil and LMIC’s named as "cardio-metabolic" and "musculoskeletal-mental" [41, 44, 45].

Considering the full set of features, the best performance was achieved for RF-CC. SVM-based transformation methods performed worse than both adaptive and transformation RF-based methods. Decision trees have already been indicated as a good method for multiple-disease classification over SVM-based methods, and our results support the choice of RF-based classifiers for multimorbidity prediction [14]. The analysis for the different feature sets confirmed that RF-based classifiers should be preferred over SVM-based classifiers from 15 predictors, selected by BR+IG. Despite the assumption of independence between diseases, RF-BR was consistently among the best predictors, with the advantage of easy implementation and scalability, However, RF-CC should be considered if subset accuracy is the most important metric, i.e. when we want to achieve the best performance regarding the accurate identification of all diseases a patient may have. The performance of the CC can decrease significantly for a dataset with a high number of labels and high dependency or cardinality, so more studies are needed for evaluation of this classifier for multimorbidity prediction [46, 47].

BMI combined with age, sex, waist-hip ratio, systolic and diastolic BP, sleep symptom and sleeping problem composed the set of eight best predictors, for MTV-RF, according variable importance. They also had the highest information gains, which was expected given that they are measures based on decision tree algorithms. Such features, found to carry the highest importance, are related to chronic diseases and multimorbidity. Sex and age are commonly reported as risk factors for multimorbidity, BMI, BP, and waist-hip ratio are also pointed out as a risk factor, especially for cardio-metabolic multimorbidities, and sleep disturbances and multimorbidity have been related previously, in particular associated with neuropsychiatric and musculoskeletal conditions [8–10, 48–50].

Our study has some limitations. Because this is a cross-sectional study, it is not possible to identify a well-established cause-effect relationship between the variables. In general, ML methods use features to predict a response, but they may not be causal factors, so the directionality between predictor and response is difficult to establish. It should be noted that the importance of the variables found is limited to the model and population studied, using other algorithms may change the relevance of the variables. Therefore, although ML provides some insights into risk factors, it is not a conclusive analysis for this purpose.

Despite these limitations, our study is the first to present a ML approach to solve the main problems in multimorbidity prediction in a scenario where the number of chronic diseases is the most used outcome in predictions. The ML methodology used and web application developed are comprehensive, and can be applied to any clinical field in which multiple outcomes are considered. Starting from a large sample size, which makes the ML process more robust, we show that it is possible, from a small set of features, that are easy to collect in clinical practice, to build a feasible tool for multimorbidity prediction.

## Supporting information

S1 Appendix (PDF)

## References

[1] Barnett K, Mercer SW, Norbury M, Watt G, Wyke S, Guthrie B. Epidemiology of multimorbidity and implications for health care, research, and medical education: a cross-sectional study. Lancet. 2012;380:37–43. doi: 10.1016/S0140-6736(12)60240-2 22579043

[2] Chatterji S, Byles J, Cutler D, et al. Health, functioning, and disability in older adults: present status and future implications. Lancet. 2015;385:563–575. doi: 10.1016/S0140-6736(14)61462-8 25468158 PMC4882096

[3] Guimarães RM, Andrade FCD. Healthy life-expectancy and multimorbidity among older adults: Do inequality and poverty matter? Archives of Gerontology and Geriatrics. 2020; 90:104157. doi: 10.1016/j.archger.2020.104157 32585554

[4] Palmer K, Marengoni A, Forjaz MJ, Jureviciene E, Laatikainen T, Mammarella F. et al. Multimorbidity care model: Recommendations from the consensus meeting of the Joint Action on Chronic Diseases and Promoting Healthy Ageing across the Life Cycle (JA-CHRODIS). Health Policy, 2018. 122:4–11. doi: 10.1016/j.healthpol.2017.09.006 28967492

[5] Hunter DJ, Reddy KS. Noncommunicable diseases. N Engl J Med. 2013. 369: 1336–1343. doi: 10.1056/NEJMra1109345 24088093

[6] Hay Simon I., et al. Global, regional, and national disability-adjusted life-years (DALYs) for 333 diseases and injuries and healthy life expectancy (HALE) for 195 countries and territories, 1990–2016: a systematic analysis for the Global Burden of Disease Study 2016. The Lancet. 390.2017: 1260–1344. 10.1016/S0140-6736(17)32130-X PMC5605707 28919118

[7] Afshar S, Roderick PJ, Kowal P, Dimitrov BD, Hill AG. Multimorbidity and the inequalities of global ageing: a cross-sectional study of 28 countries using the World Health Surveys. BMC Public Health. 2015: 15, 776. doi: 10.1186/s12889-015-2008-7 26268536 PMC4534141

[8] Abebe F, Schneider M, Asrat B, Ambaw F. Multimorbidity of chronic non-communicable diseases in low-and middle-income countries: A scoping review. Journal of comorbidity. 2020. 10: 2235042X20961919. doi: 10.1177/2235042X20961919 33117722 PMC7573723

[9] Nunes BP, Batista SRR, Andrade FBD, Souza Junior PRBD, Lima-Costa MF, Facchini LA. Multimorbidity: the Brazilian longitudinal study of aging (ELSI-Brazil). Revista de Saude publica. 2018. 52:10. 10.11606/s1518-8787.2018052000637 30379288 PMC6254906

[10] Melo LAD, Lima KCD. Prevalence and factors associated with multimorbidities in Brazilian older adults. Ciência & Saúde Coletiva. 2020. 25:3869–3877. 10.1590/1413-812320202510.34492018 32997019

[11] Panch T, Szolovits P, Atun R. Artificial intelligence, machine learning and health systems. Journal of global health. 2018:8(2). doi: 10.7189/jogh.08.020303 30405904 PMC6199467

[12] Hassaine A, Salimi-Khorshidi G, Canoy D, Rahimi K. Untangling the complexity of multimorbidity with machine learning. Mechanisms of ageing and development. 2020;190, 111325. doi: 10.1016/j.mad.2020.111325 32768443 PMC7493712

[13] Majnarić LT, Babič F, O’Sullivan S, Holzinger A. AI and big data in healthcare: towards a more comprehensive research framework for multimorbidity. Journal of Clinical Medicine. 2021. 10(4), 766. doi: 10.3390/jcm10040766 33672914 PMC7918668

[14] Zufferey D, Hofer T, Hennebert J, Schumacher M, Ingold R, Bromuri S. Performance comparison of multi-label learning algorithms on clinical data for chronic diseases. Computers in biology and medicine. 2015; 65:34–43. doi: 10.1016/j.compbiomed.2015.07.017 26275389

[15] Gibaja E, Ventura S. Multi‐label learning: a review of the state of the art and ongoing research. Wiley Interdisciplinary Reviews: Data Mining and Knowledge Discovery. 2014;4(6):411–444. 10.1002/widm.1139

[16] Pereira RB, Carvalho APD, Zadrozny B, Merschmann LHDC. Information gain feature selection for multi-label classification. 2015. Journal of Information and Data Management 6.1;48–48

[17] Charte F, Rivera AJ, del Jesus MJ, Herrera F. Addressing imbalance in multilabel classification: Measures and random resampling algorithms. Neurocomputing. 2015 163(0):3–16. 10.1016/j.neucom.2014.08.091

[18] Schmidt MI, Duncan BB, Mill JG, et al. Cohort profile: longitudinal study of adult health (ELSA-Brasil). Int J Epidemiol. 2015;44(1):68–75. doi: 10.1093/ije/dyu027 24585730 PMC4339754

[19] Aquino EM, Barreto SM, Bensenor IM, et al. Brazilian longitudinal study of adult health (ELSA-Brasil): objectives and design. Am J Epidemiol. 2012;175(4):315–324. doi: 10.1093/aje/kwr294 22234482

[20] OMS. Organização Mundial da Saúde. Diet, nutrition and the prevention of chronic diseases: report of a joint WHO/FAO expert consultation. Geneva: World Health Organization technical report series 916; 2003. Disponível em: <http://apps.who.int/iris/bitstream/10665/42665/1/WHO_TRS_916.pdf> 12768890

[21] World Health Organization: Definition and Diagnosis of Diabetes Mellitus and Intermediate Hyperglycaemia: Report of a WHO/IDF Consulation. Geneva: WHO; 2006.

[22] American Diabetes Association. 2. Classification and diagnosis of diabetes: standards of medical care in diabetes-2018. Diabetes Care. 2018;41:S13–27. 10.2337/dc18-S002 29222373

[23] Nunes MAA et al. Adaptação transcultural do CIS-R (Clinical Interview Schedule-Revised version) para o português no Estudo Longitudinal de Saúde do Adulto (ELSA). Revista HCPA. 2011. 31: 487–490, 2011.

[24] Benseñor IM, Lotufo PA, Pereira AC, et al. Validation of a questionnaire for the diagnosis of headache in an outpatient clinic at a university hospital. Arq Neuropsiquiatr. 1997;55:364–369. 10.1590/S0004-282X1997000300003 9629350

[25] Mill JG et al. Medical assessments and measurements in ELSA-Brasil. Rev Saude Publica. 2013. 47:54–62 10.1590/S0034-8910.2013047003851 24346721

[26] Ahmadi B, Alimohammadian M, Yaseri M, Majidi A, Boreiri M, Islami F, et al. Multimorbidity: epidemiology and risk factors in the Golestan cohort study, Iran: a cross-sectional analysis. Medicine. 2016; 95(7). doi: 10.1097/MD.0000000000002756 26886618 PMC4998618

[27] Nusinovici S, Tham YC, Yan MYC, Ting DSW, Li J, Sabanayagam C, et al. Logistic regression was as good as machine learning for predicting major chronic diseases. Journal of clinical epidemiology. 2020;122:56–69. doi: 10.1016/j.jclinepi.2020.03.002 32169597

[28] Olivera AR, Roesler V, Iochpe C, Schmidt MI, Vigo Á, Barreto SM, et al. Comparison of machine-learning algorithms to build a predictive model for detecting undiagnosed diabetes-ELSA-Brasil: accuracy study. Sao Paulo Medical Journal.2017. 135(3), 234–246. doi: 10.1590/1516-3180.2016.0309010217 28746659 PMC10019841

[29] Santana NMT, Mill JG, Velasquez-Melendez G, et al. Consumption of alcohol and blood pressure: results of the ELSA-Brasil study. PLoS One. 2018;13(1):e0190239. doi: 10.1371/journal.pone.0190239 29309408 PMC5757983

[30] Bensenor IM, Griep RH, Pinto KA, et al. Rotinas de organização de exames e entrevistas no centro de investigação ELSA-Brasil. Rev Saúde Pública. 2013;47(2):37–47. 10.1590/S0034-8910.2013047003780 24346719

[31] Chor D, Cardoso LO, Nobre AA, Griep RH, Fonseca MDJM, Giatti L, et al. Association between perceived neighbourhood characteristics, physical activity and diet quality: results of the Brazilian Longitudinal Study of Adult Health (ELSA-Brasil). BMC Public Health.2016 16(1), 1–11. 10.1186/s12889-016-3447-5 27506819 PMC4977638

[32] Schafer I, Kaduszkiewicz H, Wagner HO, Schon G, Scherer M, Bussche H. Reducing complexity: a visualisation of multimorbidity by combining disease clusters and triads. BMC Public Health2014;14:1285. doi: 10.1186/1471-2458-14-1285 25516155 PMC4301832

[33] Segal M, Xiao Y. Multivariate random forests. Wiley interdisciplinary reviews: Data mining and knowledge discovery. 2011; 1:80–87. 10.1002/widm.12

[34] Probst P, Au Q, Casalicchio G, Stachl C, Bischl B. Multilabel classification with R package mlr. arXiv preprint arXiv. 2017;1703.08991. 10.32614/RJ-2017-012

[35] Charte F, Charte D. Working with Multilabel Datasets in R: The mldr Package. R J. 2015;7(2):149. 10.32614/RJ-2015-027

[36] Lusa L. Joint use of over-and under-sampling techniques and cross-validation for the development and assessment of prediction models. BMC bioinformatics.2015; 16(1):1–10. doi: 10.1186/s12859-015-0784-9 26537827 PMC4634915

[37] Tantithamthavorn C, Hassan AE, Matsumoto K. The impact of class rebalancing techniques on the performance and interpretation of defect prediction models. IEEE Transactions on Software Engineering, 2018.46(11):1200–1219. 10.1109/TSE.2018.2876537

[38] Spolaôr N, Cherman EA, Monard MC, Lee HD. A comparison of multi-label feature selection methods using the problem transformation approach. Electronic Notes in Theoretical Computer Science, 2013;292:135–151. 10.1016/j.entcs.2013.02.010

[39] Stirland LE, González-Saavedra L, Mullin DS, Ritchie CW, Muniz-Terrera G, Russ TC. Measuring multimorbidity beyond counting diseases: systematic review of community and population studies and guide to index choice. Bmj. 2020; 368. doi: 10.1136/bmj.m160 32071114 PMC7190061

[40] Macinko J, Andrade FC, Nunes BP, Guanais FC. Primary care and multimorbidity in six Latin American and Caribbean countries. Revista Panamericana de Salud Publica. 2019;43, e8. doi: 10.26633/RPSP.2019.8 31093232 PMC6393736

[41] Rzewuska M, de Azevedo-Marques JM, Coxon D, Zanetti ML, Zanetti ACG, Franco LJ, et al. Epidemiology of multimorbidity within the Brazilian adult general population: Evidence from the 2013 National Health Survey (PNS 2013). PloS one. 2017; 12(2), e0171813. doi: 10.1371/journal.pone.0171813 28182778 PMC5300133

[42] Carvalho JND, Roncalli ÂG, Cancela MDC, Souza DLBD. Prevalence of multimorbidity in the Brazilian adult population according to socioeconomic and demographic characteristics. PloS one. 2017;12(4):e0174322. doi: 10.1371/journal.pone.0174322 28384178 PMC5383049

[43] Wang YP, Chiavegatto-Filho AD, Benseñor IM, Viana MC, Andrade LH. Patterns of multimorbidity in the general population of São Paulo, Brazil: a retrospective observational study. The Lancet. 2014; 384, S22. 10.1016/S0140-6736(14)61885-7

[44] Peres MFP, Queiroz LP, Rocha-Filho PS, Sarmento EM, Katsarava Z, Steiner TJ. Migraine: a major debilitating chronic non-communicable disease in Brazil, evidence from two national surveys. The journal of headache and pain. 2019;20(1):1–6. 10.1186/s10194-019-1036-6 31370786 PMC6734239

[45] Garin N, Koyanagi A, Chatterji S, Tyrovolas S, Olaya B, Leonardi M, et al. Global multimorbidity patterns: a cross-sectional, population-based, multi-country study. J Gerontol A Biol Sci Med Sci. 2016;71(2):205–14. doi: 10.1093/gerona/glv128 26419978 PMC5864156

[46] Montanes E, Senge R, Barranquero J, Quevedo JR, del Coz JJ, Hüllermeier E. Dependent binary relevance models for multi-label classification. Pattern Recognition.2014;47(3):1494–1508. 10.1016/j.patcog.2013.09.029

[47] Luaces Oscar, et al. Binary relevance efficacy for multilabel classification. Progress in Artificial Intelligence 1.4. 2012; 303–313. 10.1007/s13748-012-0030-x

[48] Kivimäki M, Kuosma E, Ferrie JE, Luukkonen R, Nyberg ST, Alfredsson L, et al. Overweight, obesity, and risk of cardiometabolic multimorbidity: pooled analysis of individual-level data for 120 813 adults from 16 cohort studies from the USA and Europe. The Lancet Public Health. 2017; 2(6): e277–e285. doi: 10.1016/S2468-2667(17)30074-9 28626830 PMC5463032

[49] Lu Y, Liu S, Qiao Y, Li G, Wu Y, Ke C. Waist-to-height ratio, waist circumference, body mass index, waist divided by height0. 5 and the risk of cardiometabolic multimorbidity: a national longitudinal cohort study. Nutrition, Metabolism and Cardiovascular Diseases. 2021. doi: 10.1016/j.numecd.2021.05.026 34226121

[50] Sindi S, Pérez LM, Vetrano DL, Triolo F, Kåreholt I, Sjöberg L, et al. Sleep disturbances and the speed of multimorbidity development in old age: results from a longitudinal population-based study. BMC medicine.2020; 18(1): 1–10. 10.1186/s12916-020-01846-w 33280611 PMC7720467

