# Supplementary material for: Comparing machine learning algorithms for multimorbidity prediction: An example from the Elsa-Brasil study
Source: PLoS One. 2022 Oct 7;17(10):e0275619. doi: 10.1371/journal.pone.0275619 (PMC9543987; doi:10.1371/journal.pone.0275619)
Supplement: S1 Appendix — (PDF) [file pone.0275619.s001.pdf]

Appendix S1

Contents

1- **SMethods 1: Hyperparameter tuning**
2- **SResults 1: Resample algorithm**
3- **SResults 2: Example of a tree for a multivariate random forest classifier**
4- **SResults 3: Brief web application tutorial**
5- **References**

1- SMethods 1: Hyperparameter tuning

For parameter tuning, we use random search, which has a lower computational cost than grid search, and identify parameter values that lead to the best performance through nested cross-validation.

For the transformation methods, we use random forest (RF) and support vector machine (SVM) as base classifiers. The transformation methods based on SVM were trained using a radial kernel. The data were scaled to zero mean and unit variance internally, per default. The hyperparameters cost and γ were set using nested 3-fold cross-validation. The R packages mlr and e1701 were used for the implementation.

For the transformation methods based on RF, the hyperparameters ntree, mtry, and nodesize were set using nested 3-fold cross-validation. The Gini index was used as the splitting rule and the bootstrap resamples were left at their default values (drawn without replacement with the size of the sample 0.632 times the sample size). The R packages mlr and randomForestSRC were used.

The multivariate random forest was also trained through the R packages mlr and randomForestSRC, and the hyperparameters ntree, mtry, and nodesize were set using nested 3-fold cross-validation. The other parameters were used at their default values: the splitting rule was the multivariate normalized Gini index splitting and the bootstrap resamples were drawn without replacement with the size of the sample 0.632 times the sample size.

2- SResults 1: Resample algorithm

To analyze the effect of resampling on performance, we applied the random oversample based on the IRLbl measure on multivariate random forest. The labels whose IRLbl is higher than MeanIR would be considered as minority labels. This criterion was used to define the instances that would be replicated. The results showed that the performance had a slight improvement over that found without considering resampling:

Accuracy: 0.362; Subset accuracy: 0.173; Hamming loss: 0.160; F-measure: 0.433.

3 - SResults 2: Example of trees for a multivariate random forest classifier

For simplicity, a sample size of 1340 from the study population was used, stratified according to the percentage of the diseases. The ntree, mtry, and nodesize parameters were identified following hyperparameter tuning described in SMethods 1.

Figure 1. Example of trees for the multivariate random forest with targets cancer and diabetes, with a maximum depth of eight, and that can use all the 31 features to make the splits.

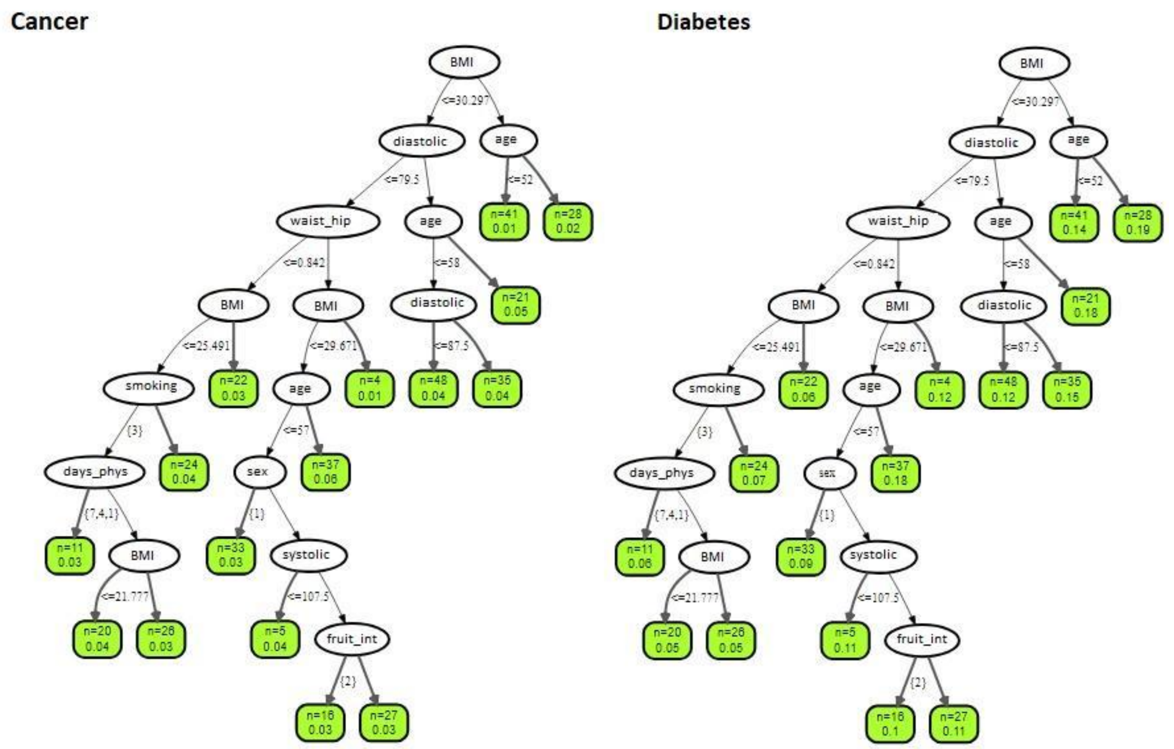

BMI: Body mass index. Smoking: 1 -Past, 2 - Current, 3- Never. Sex: 1- Female, 2 -Male. Days_phys: days of physical activity (from 1 to 7 days per week). Fruit_int: fruit intake (4- twice or more per day; 3- once a day/five to six times a week; 2-two to four times a week; and 3-once a week or less).

Figure 2. Example of trees for the multivariate random forest with targets and heart diseases and Dyslipidemia, with a maximum depth of eight, and that can use all the 31 features to make the splits.

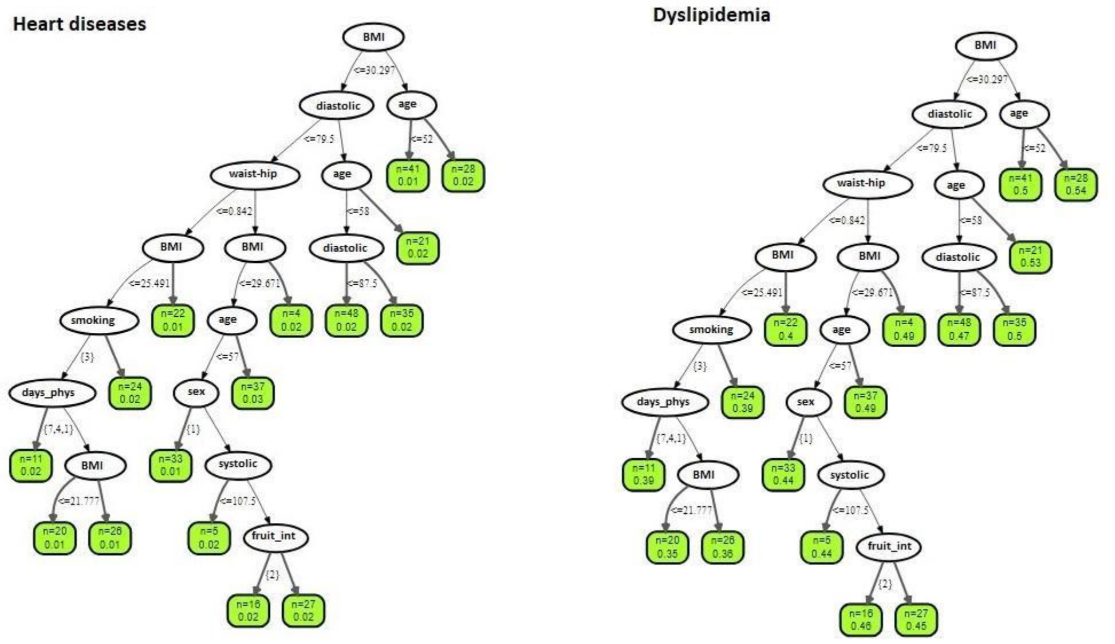

BMI: Body mass index. Smoking: 1 -Past, 2 - Current, 3- Never. Sex: 1- Female, 2 -Male. Days_phys: days of physical activity (from 1 to 7 days per week). Fruit_int: fruit intake (4- twice or more per day; 3- once a day/five to six times a week; 2-two to four times a week; and 3-once a week or less).

Figure 3. Example of trees for the multivariate random forest with targets common mental disorder and migraine, with a maximum depth of eight, and that can use all the 31 features to make the splits.

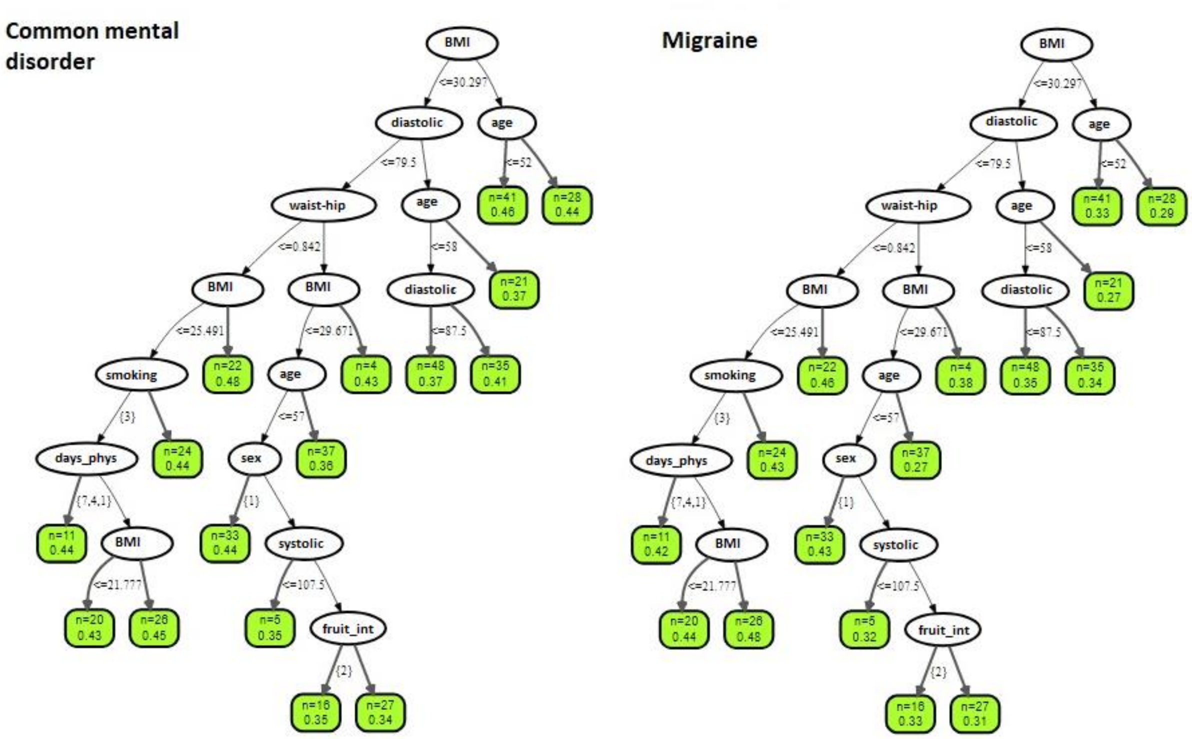

BMI: Body mass index. Smoking: 1 -Past, 2 - Current, 3- Never. Sex: 1- Female, 2 -Male. Days_phys: days of physical activity (from 1 to 7 days per week). Fruit_int: fruit intake (4- twice or more per day; 3- once a day/five to six times a week; 2-two to four times a week; and 3-once a week or less).

Figure 4. Example of trees for the multivariate random forest with targets joint problems and kidney disease, with a maximum depth of eight, and that can use all the 31 features to make the splits.

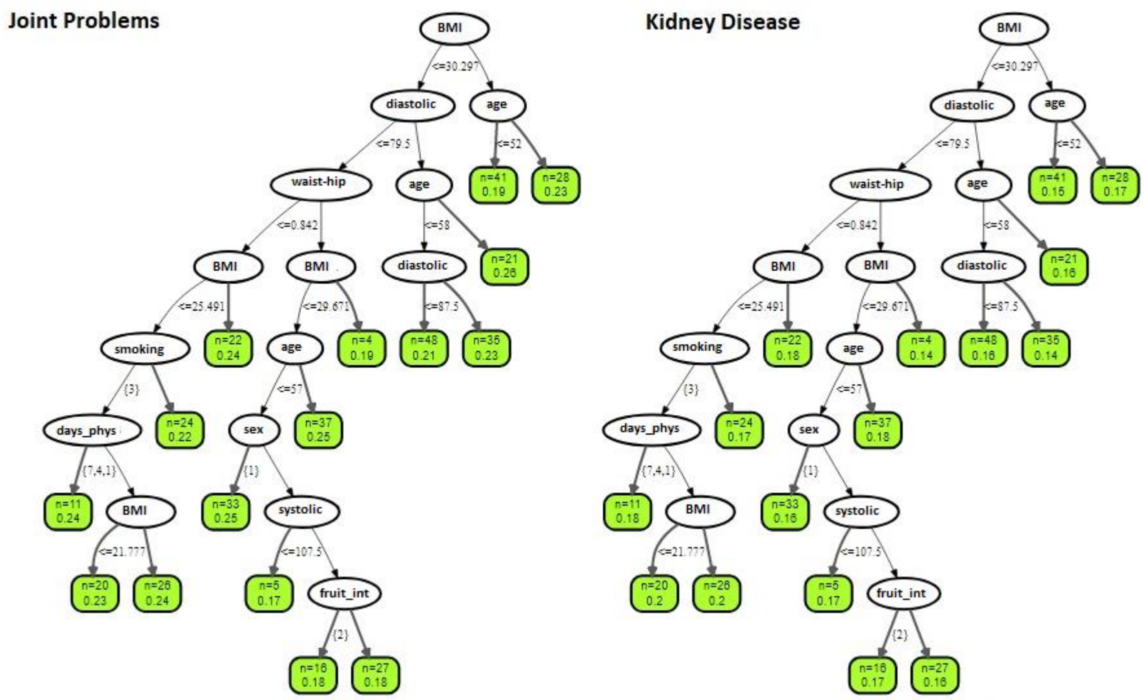

BMI: Body mass index. Smoking: 1 -Past, 2 - Current, 3- Never. Sex: 1- Female, 2 -Male. Days_phys: days of physical activity (from 1 to 7 days per week). Fruit_int: fruit intake (4- twice or more per day; 3- once a day/five to six times a week; 2-two to four times a week; and 3-once a week or less).

Figure 5. Example of trees for the multivariate random forest with targets asthma and cirrhosis, with a maximum depth of eight, and that can use all the 31 features to make the splits.

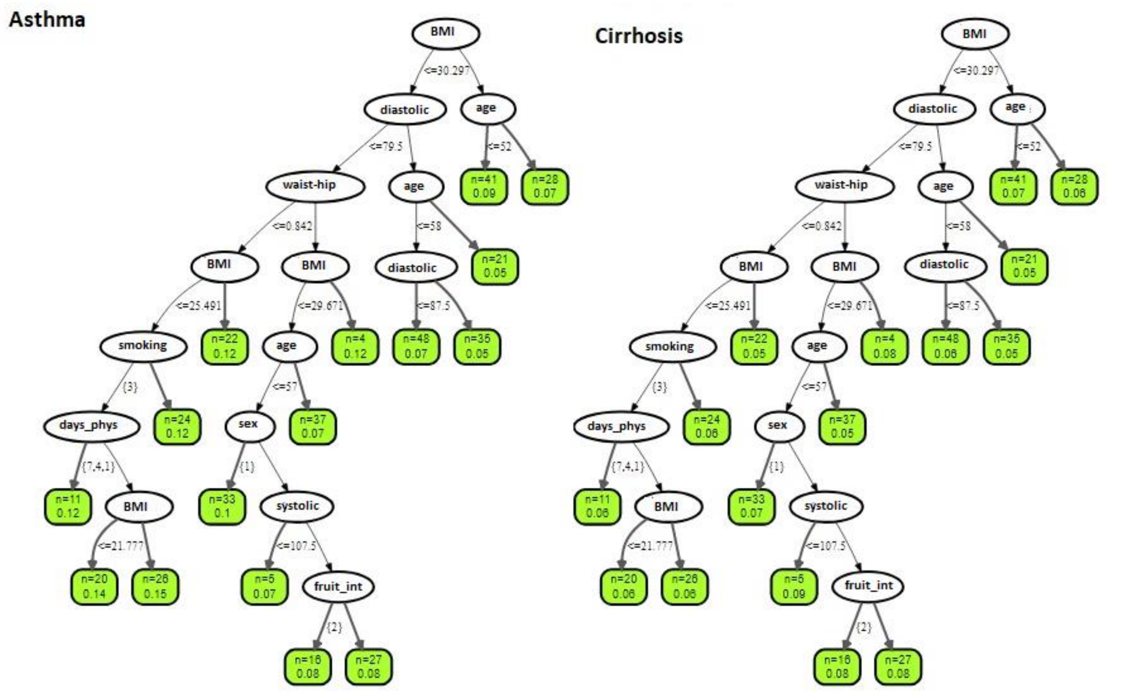

BMI: Body mass index. Smoking: 1 -Past, 2 - Current, 3- Never. Sex: 1- Female, 2 -Male. Days_phys: days of physical activity (from 1 to 7 days per week). Fruit_int: fruit intake (4- twice or more per day; 3- once a day/five to six times a week; 2-two to four times a week; and 3-once a week or less).

4 - SResults 3: Brief web application tutorial

The tool basically consists of the following steps: creating the models, checking results and performance, and using the created models to generate predictions.

There are 4 main tabs at the top of the tool:

1. **Home:** It presents an overview about the tool, and general concepts. It also provides two examples of datasets that can be used as template input files for building the models, and performing prediction.
2. **About Classifiers:** Divided into sub tabs, it presents everything that is needed to understand the features of the models presented in the tool. It is possible to return to the explanations about the models anytime, in this section.
3. **Models - Classifiers:** This tab is the section responsible for creating the models and using them.
4. **Information:** Presents information about the construction of the tool, contact information, and useful references.

Each of the four main tabs and their functionality are detailed below

1. Home: It presents an overview about the tool, and general concepts. For the user to become familiar with the usability of the web application, it also provides two examples of datasets that can be used as template input files for building the models, and performing prediction.

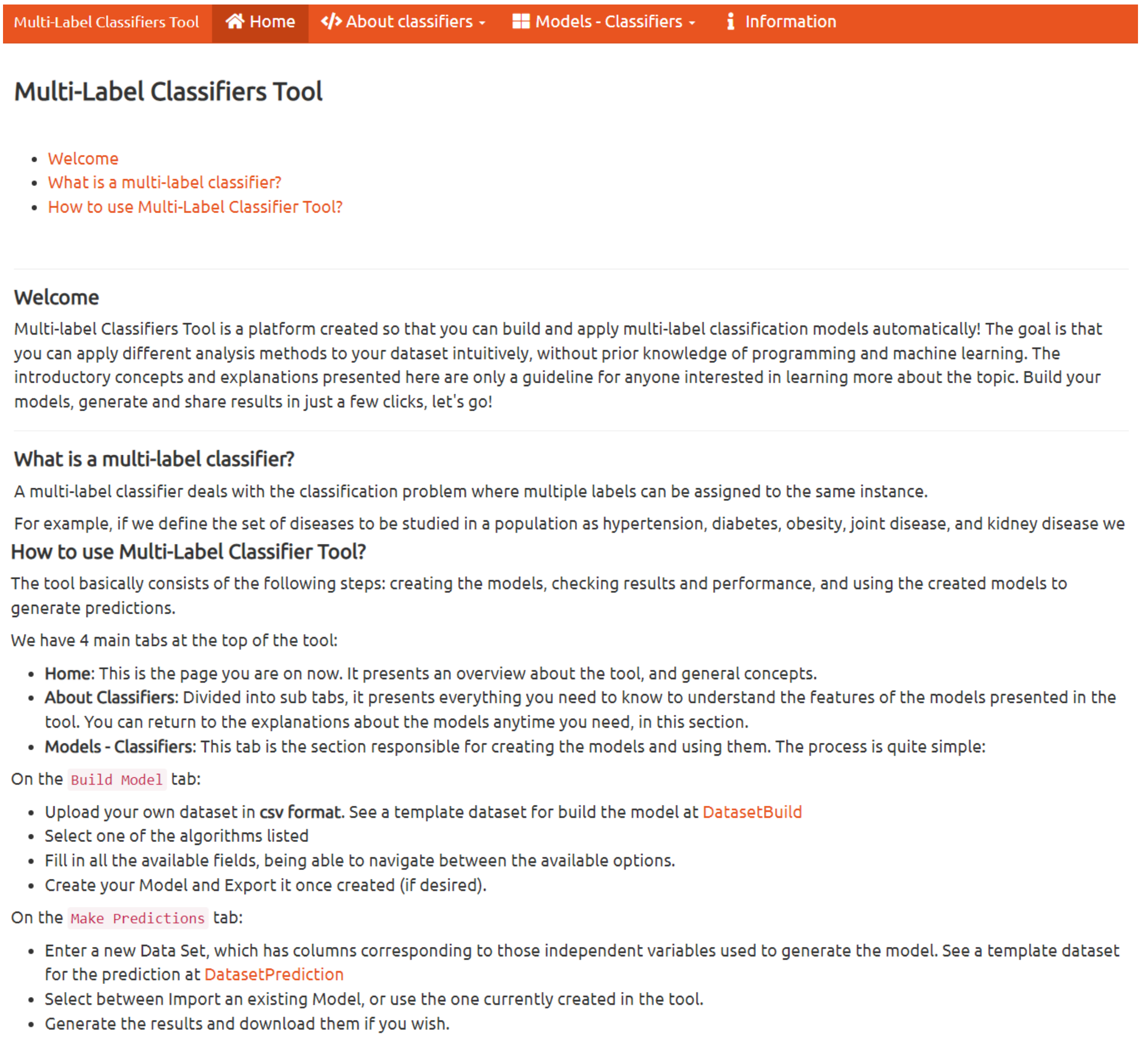

The examples of datasets that can be used as template input files for building the models, and performing prediction can be accessed through the two links, called *DatasetBuild* and *DatasetPrediction*, as shown in the figure above, available in the web application at:

Home -> How to use the Multi-Label classifier tool

The links will take the user directly to the files, which are stored on github, in the web application's documentation directory. They can be downloaded separately, as follows:

DatasetBuild - This link will take the user to the page with the file, as shown in the picture below. Just click on the Raw button, marked on the picture, and a new page will open with the file data. By clicking with the right mouse button anywhere on the page, an option will appear for the user to save the file as csv.

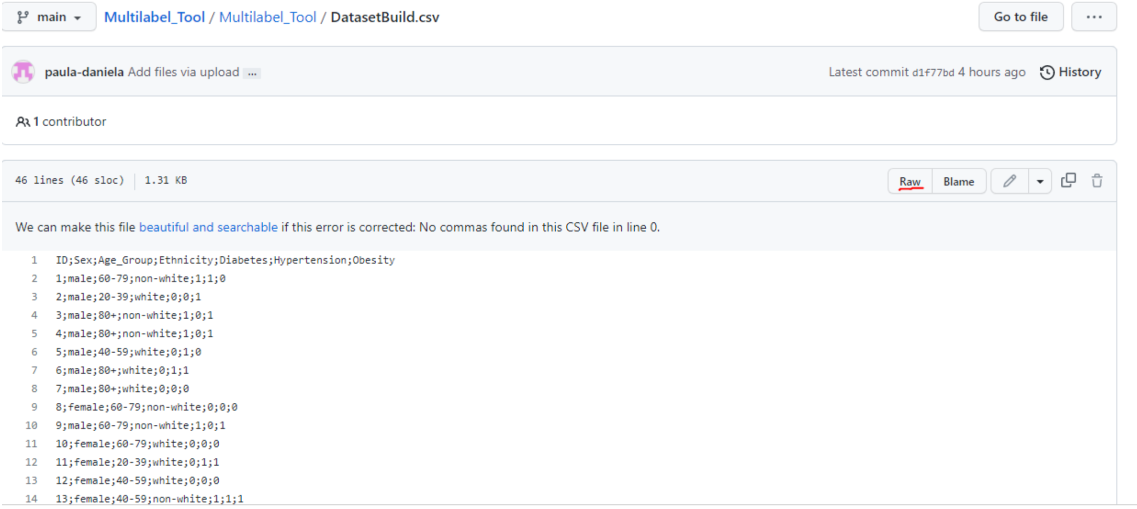

DatasetPrediction - This link will take the user to the page with the file, as shown in the picture below. The same procedure for download DatasetBuild can be used.

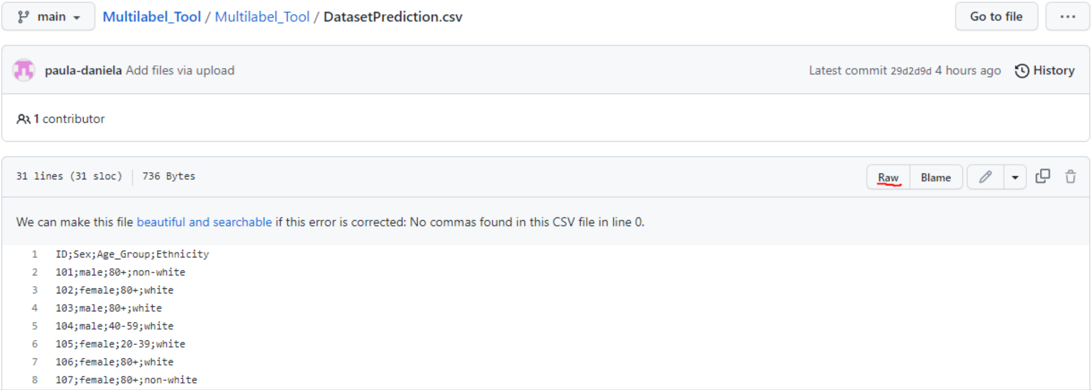

2. About Classifiers: Provides information about the models available, preprocessing, such as standardization and missing data treatment, hyperparameter tuning, resampling and validation, model evaluation and feature selection.

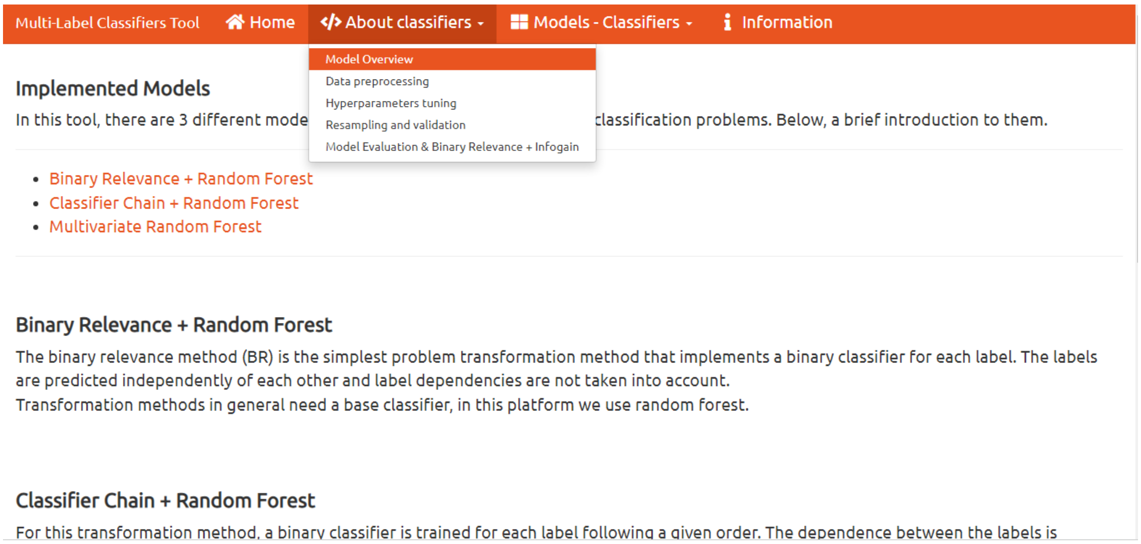

3. Models - Classifiers: This is the section responsible for creating the models and using them. There are two tabs in this section: Build Model tab - Where it is possible to build the model and export it for further use, and Make Predictions tab - Where it is possible to use the built model, or import a previous model to make predictions on a new data set. The process is quite simple:

i - On the Build Model tab:

- Upload the dataset in csv format (Here the user can upload the ***DatasetBuild*** dataset, downloaded earlier).
- Select one of the algorithms listed
- Fill in all the available fields, being able to navigate between the available options.
- Create the Model and Export it once created (if desired).

The figures below show the results of building the model using DatasetBuild data

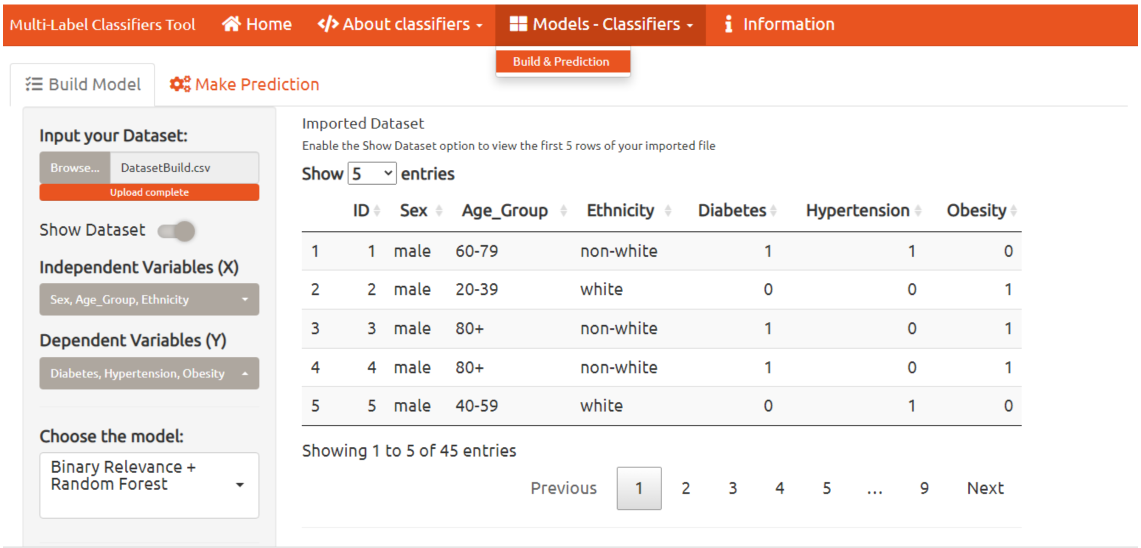

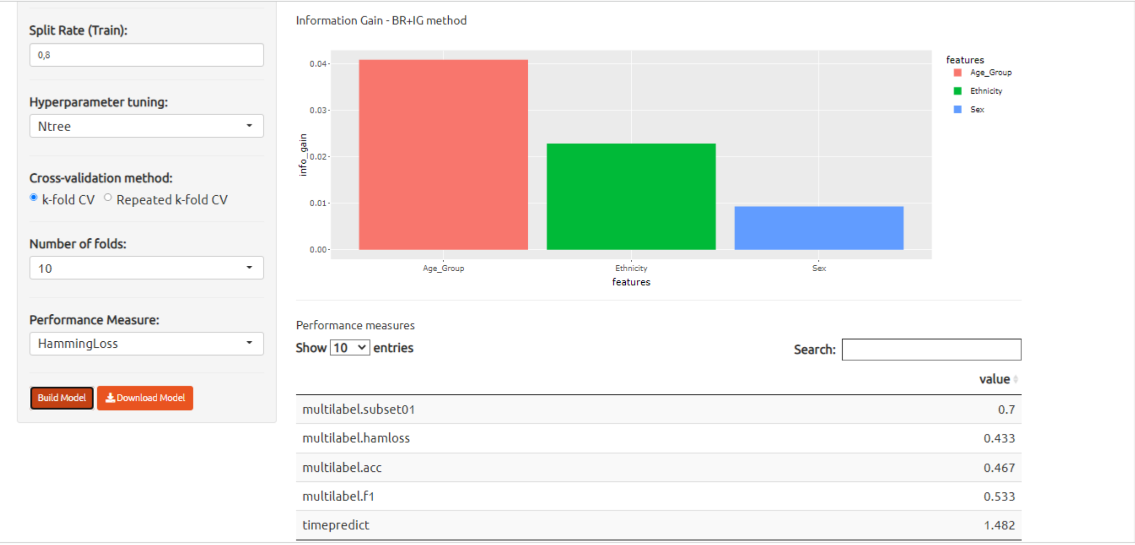

ii - On the Make Predictions tab:

- Enter a new Data Set, which has the same columns corresponding to those used to generate the model (Here the user can upload the ***DatasetPredicion*** dataset, downloaded earlier).
- Select between Import an existing Model, or use the one currently created in the tool.
- Generate the results and download them if desired.

The figures below show the results of prediction using DatasetPrediction data

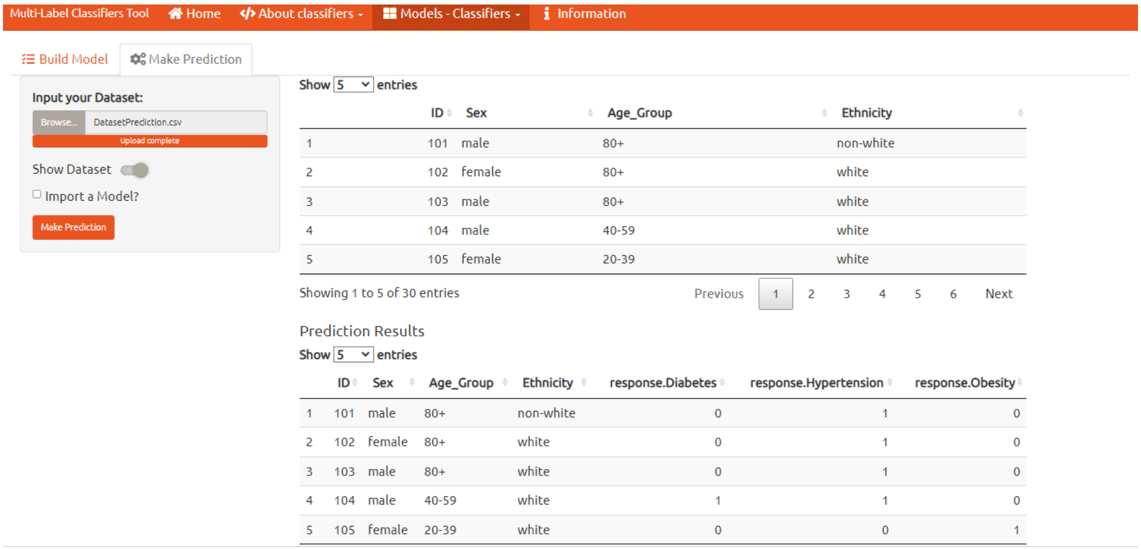

<u>4 - Information:</u> Presents information about the construction of the tool, contact information, and useful references.

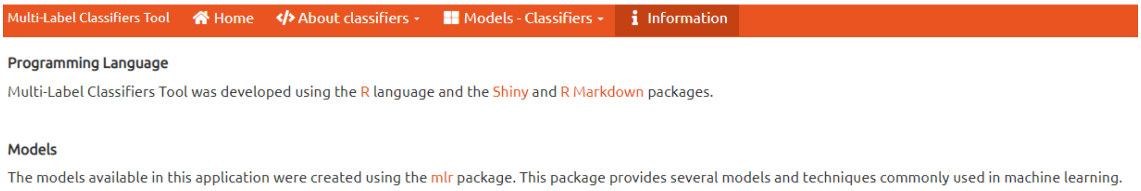

5 - References

[1] Probst, P., Au, Q., Casalicchio, G., Stachl, C., & Bischl, B. (2017). Multilabel classification with R package mlr. *arXiv preprint arXiv:1703.08991*.

[2] Segal, M., & Xiao, Y. (2011). Multivariate random forests. *Wiley interdisciplinary reviews: Data mining and knowledge discovery*, *1*(1), 80-87.

[3] Gibaja, E., & Ventura, S. (2014). Multi-label learning: a review of the state of the art and ongoing research. *Wiley Interdisciplinary Reviews: Data Mining and Knowledge Discovery*,*4*(6), 411-444.

[4] F. Charte, A. J. Rivera, M. J. del Jesus, and F. Herrera. Addressing imbalance in multilabel classification: Measures and random resampling algorithms. Neurocomputing, 163(0):3–16, 2015.
